# Comparison of [^18^F]FDG and [^18^F]PSMA-1007 PET/CT in the Evaluation of Muscle-Invasive Bladder Cancer: A Pilot Feasibility Study

**DOI:** 10.1007/s11307-026-02085-w

**Published:** 2026-03-23

**Authors:** Natália Dalsenter Avilez, Ricardo N. Tineo, Juliano Tomé Rodrigues, Arthur Degani Ottaiano, Helena P. A. Saito, Bárbara Juarez Amorim, José Barreto Campello Carvalheira, Leonardo O. Reis, Celso Dario Ramos

**Affiliations:** 1 Faculty of Medical Sciences, UroScience, University of Campinas (UNICAMP), São Paulo, Brazil; 2 Division of Nuclear Medicine, Department of Radiology and Oncology, Faculty of Medical Sciences, University of Campinas (UNICAMP), São Paulo, Brazil; 3 Division of Oncology, Department of Radiology and Oncology, Faculty of Medical Sciences, University of Campinas (UNICAMP), São Paulo, Brazil; 4 Cancer Theranostics Innovation Center (CancerThera), Universidade Estadual de Campinas (UNICAMP), Campinas, São Paulo, Brazil; 5 ImmunOncology, Pontifical Catholic University of Campinas (PUC-Campinas), Campinas, São Paulo, Brazil; 6 INCT UroGen, National Institute of Science, Technology and Innovation in Genitourinary Cancer, Campinas, São Paulo, Brazil

**Keywords:** PET/CT imaging, [^18^F]PSMA-1007, [^18^F]FDG, Muscle-invasive bladder urothelial carcinoma, Theranostic

## Abstract

**Purpose:**

To compare the diagnostic performance of [^18^F]FDG and [^18^F]PSMA‑1007 PET/CT for detecting primary tumors, regional lymph node involvement, and distant metastases in recently diagnosed muscle‑invasive bladder cancer (MIBC).

**Methods:**

Prospective single‑center cohort of six patients (ages 57–82). Both PET/CTs were acquired within 30 days under EANM/SNMMI-conformant protocols, with blinded consensus readings and post-diuretic pelvic acquisitions.

**Results:**

[^18^F]PSMA‑1007 identified 15 lesions versus 14 with [^18^F]FDG. Primary bladder lesions were detected in 5 of 6 patients, compared to 3 of 6 patients. Both tracers detected nodal metastases in three patients and bone metastases in one. An [^18^F]FDG‑avid pulmonary lesion near the spleen was not detected with [^18^F]PSMA‑1007 owing to physiological splenic uptake.

**Conclusion:**

Both tracers showed comparable sensitivity for metastatic disease. The hepatobiliary clearance of [^18^F]PSMA‑1007 improved visualization of intravesical disease, supporting its use in staging and potential theranostic strategies in selected MIBC patients. Such real-world findings inform refinements for future study procedures, logistics, and methodological design, which are essential for minimizing research waste by identifying potential problems early.

Trial Registration: Not applicable.

**Graphical Abstract:**

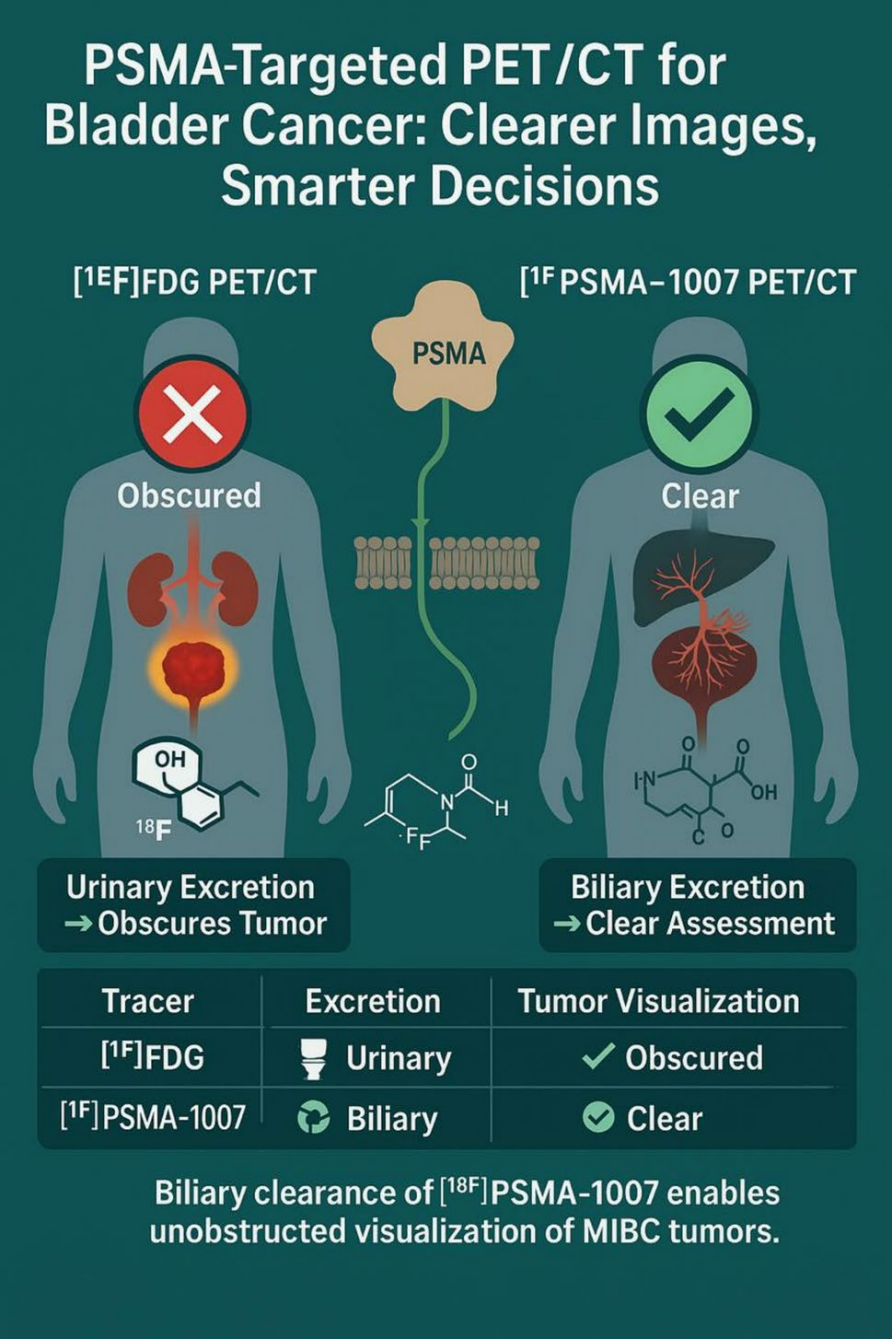

## Introduction

Muscle-invasive bladder cancer (MIBC) is a highly aggressive malignancy with significant rates of metastasis and recurrence, necessitating effective imaging strategies for accurate staging and disease monitoring [1]. Exams such as computed tomography (CT) and magnetic resonance imaging (MRI) are used in disease staging; however, they are limited in their evaluation of lymph nodes. This is because the assessment of lymph node metastases based solely on size is limited; both CT and MRI are unable to identify metastases in normal-sized or minimally enlarged lymph nodes. Pelvic lymph nodes measuring more than 8 mm and abdominal lymph nodes measuring more than 10 mm in maximum short-axis diameter, as detected by CT or MRI, should be considered pathologically enlarged [2]. However, the specificity is also low because lymph node enlargement may be due to benign disease.

In this context, metabolic assessment tests are a valuable and effective tool for this evaluation. Positron emission tomography/computed tomography (PET/CT) with [^18^F]fluorodeoxyglucose ([^18^F]FDG) is widely used in oncological imaging, including the evaluation of MIBC. However, the primary renal excretion of [^18^F]FDG often leads to intense radioactive accumulation in the urinary tract, which can obscure bladder lesions and complicate image interpretation, even with diuretic administration [3]. In this setting, [^18^F]FDG PET/CT cannot be proposed as a stand-alone imaging procedure but can be proposed as a complementary tool.

Prostate-specific membrane antigen (PSMA) expression has been observed in the neo-angiogenesis of various non-prostatic malignancies, including urothelial carcinoma, suggesting a potential role for PSMA-targeted imaging in MIBC. Among PSMA-targeting radiotracers, [^18^F]PSMA-1007 stands out due to its predominant hepatobiliary clearance, which minimizes urinary excretion and may improve lesion detection within the bladder. Despite the growing interest in PSMA-based imaging, the comparative efficacy of [^18^F]PSMA-1007 and [^18^F]FDG PET/CT in MIBC remains unclear [4].

This study aimed to evaluate the feasibility of comparing the diagnostic performance of [^18^F]FDG and [^18^F]PSMA-1007 PET/CT in patients with recently diagnosed MIBC (stage T2 or higher) prior to systemic treatment, assessing their ability to detect residual bladder disease after transurethral resection of bladder tumor (TUR-BT), regional lymph node involvement, and distant metastases.

## Materials and Methods

Following approval by the Ethics Committee of the University of Campinas (UNICAMP) (CAAE: 60,267,622.0.0000.5404), we conducted a prospective cohort study including patients with locally advanced MIBC, confirmed by TUR-BT and staged as T2 or higher.

The inclusion criteria were patients aged > 40 years, under follow-up at the Oncology Outpatient Clinic of the Hospital de Clínicas, UNICAMP, with histopathologically confirmed urothelial carcinoma of the bladder showing invasion of the *muscularis propria*, and presenting with active disease (i.e., newly diagnosed before treatment initiation). No restrictions were applied regarding ethnicity or sex.

Exclusion criteria included a history of other primary or concurrent malignancies, active infection, or any condition potentially associated with inflammatory reactions (e.g., recent surgery, vaccination, or trauma within the last 3 months). Eligible patients were invited to participate, provided written informed consent, and subsequently underwent both [^18^F]FDG and [^18^F]PSMA-1007 PET/CT examinations.

[^18^F]FDG PET/CT scans were performed following the European Association of Nuclear Medicine (EANM) procedure guidelines for tumor imaging with [^18^F]FDG [5]. Patients were instructed to fast (except for water) for 6 h before [^18^F]FDG PET/CT injection. Blood glucose levels were checked before the injection and all patients presented glucose levels below 180 mg/dl. Patients were well-hydrated both before and during the study period. Voiding was asked immediately before imaging acquisition.

Full-body images (from head to upper thighs) were acquired 60 min after intravenous (IV) administration of 4.4 MBq/kg (0.12 mCi/kg) of [^18^F]FDG (Center of Molecular Research – CMR, Campinas, Brazil) in patients with blood glucose levels below 180 mg/dL, using a Biograph True-Point mCT 40 PET/CT scanner (Siemens Medical Solutions Inc., Knoxville, Tennessee, USA). No radiological contrast was used. CT scans were acquired at 120–140 kV, 120 mA, with a rotation time of 0.8 s, and a slice thickness of 2.1 mm. PET images were acquired in three-dimensional mode using 1.5 min/bed position.

PSMA-PET/CT scans were performed according to the joint EANM and Society of Nuclear Medicine and Molecular Imaging (SNMMI) procedure guidelines for prostate cancer imaging [6], adapted for the tracer [^18^F]PSMA-1007. Patients were well-hydrated both before and during the study period. Voiding was asked immediately before imaging acquisition. Initial PET/CT images of the pelvic region were acquired 5 min after IV administration of 3.7 MBq/kg (0.1 mCi/kg) of [^18^F]PSMA-1007 (Center of Molecular Research – CMR, Campinas, Brazil). Full-body images (from head to upper thighs) were acquired 90 min after tracer injection using the same protocol described above for [^18^F]FDG PET/CT, except that the scan time was 2.0 min/bed position.

After each [^18^F]FDG PET/CT and [^18^F]PSMA-1007 PET/CT acquisition from the head to the upper thighs, patients were injected with 20 mg of furosemide intravenously. They then received oral hydration with 800–1000 mL of water and were instructed to urinate frequently. New PET/CT images of the pelvic region were acquired 1 h after IV injection of furosemide.

Two experienced nuclear physicians and one radiologist analyzed PET/CT images, blinded to the results of other imaging studies (consensus readings). The number and location of regions with abnormal tracer accumulation were described. The reference standard for lesion assessment was based on visual and semi-quantitative analysis using SUV values, as outlined in the EANM procedure guidelines for tumor imaging [5] and the joint EANM and SNMMI procedure guidelines for prostate cancer imaging [6]. Normal physiological uptake in reference organs, such as the liver, and blood pool was used to aid in determining whether the uptake should be considered pathological.

The data obtained from the exams were compared with the results of conventional imaging exams (CT and MRI), as well as other clinicopathological characteristics extracted from the medical records, such as clinical stage, histopathology, metastasis, treatment performed, time between diagnosis and disease progression, among others.

## Results

Six patients (five males, aged 57–82) participated in the prospective PET/CT study. The characteristics of the patients studied are presented in Table 1.

**Table 1:** Study population characteristics

Patient	Age	Sex	Stage	Histology
1	58	Male	T4N1M1	Poorly differentiated carcinoma with extensive areas of squamous differentiation
2	72	Male	T3N1M0	High-grade papillary and non-papillary carcinoma
3	57	Male	T2aN0M0	High-grade urothelial carcinoma, nest variant
4	73	Male	T4N1M1	High-grade non-papillary urothelial carcinoma
5	80	Male	T2N0M0	High-grade urothelial carcinoma
6	82	Female	T3N0M0	High-grade urothelial carcinoma

The median time between the two scans was 3.5 days (1 to 29 days). Only one patient had an interval of more than one week between the two exams due to hospitalization for hematuria, which made it impossible to perform the second exam earlier.

**Fig. 1:**
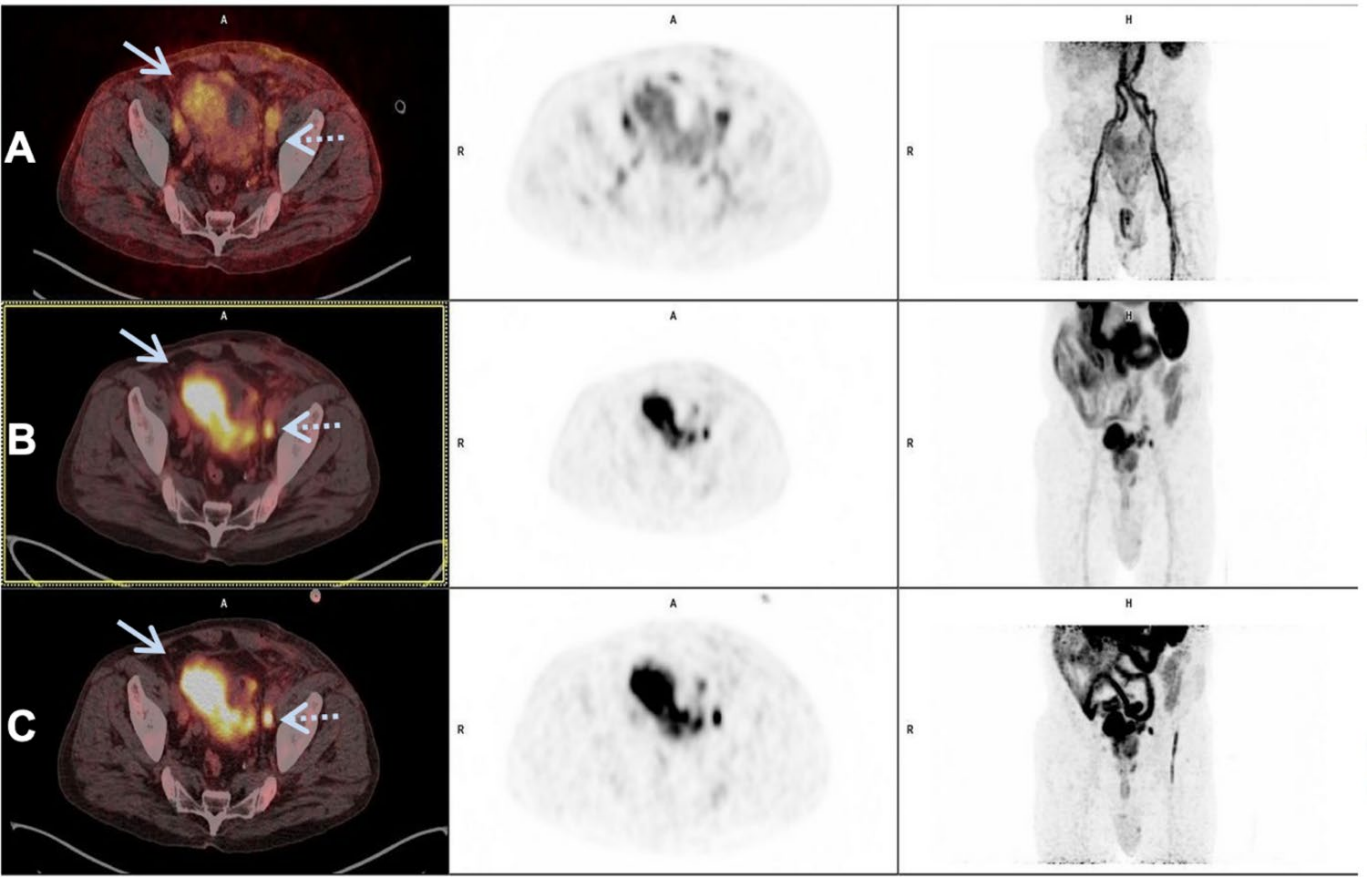
72-year-old, male, T3N1M0, high-grade papillary and non-papillary carcinoma. From left to right: axial fused PET/CT, axial PET, and maximum intensity projection [^18^F]PSMA-1007 images of a patient (patient 2, Table 1) with muscle-invasive bladder urothelial carcinoma and lymph node metastases at (**A**) 5 min, (**B**) 90 min, and (**C**) 2 h after tracer injection. Note the progressive increase in [^18^F]PSMA-1007 uptake in the bladder main lesion (solid arrow) and in a left lymph node metastasis (dotted arrow) from 5 min to 2 h after tracer injection. No significant urinary excretion of [^18^F]PSMA-1007 is seen in this patient.

[^18^F]PSMA-1007 uptake in bladder lesions and regional lymph nodes increased progressively between the images acquired at 5 min, 90 min, and 2 h (Fig. 1). Fifteen lesions were identified in the 6 patients with [^18^F]PSMA-1007, compared to 14 lesions observed with [^18^F]FDG. The comparison of [^18^F]FDG and [^18^F]PSMA-1007 PET/CT is detailed in Table 2.

**Table 2:** Comparison of lesion detectability between [^18^F]FDG and [^18^F]PSMA-1007 PET/CT and MRI findings in muscle-invasive bladder urothelial carcinoma

Patient	Time between FDG and PSMA scans (days)	MRI findings		SUVmax: primary tumor site		SUVmax: metastases		Metastasis site with greatest uptake		Number of sites detected^3^	
		Primary lesion size (cm)	Metastatic lymph nodes	FDG	PSMA	FDG	PSMA	FDG	PSMA	FDG	PSMA
**1**	4^**1**^	6.8	Not detected	Not detected	9.8	17.4	11.1	Distant lymph node	Bone	4	5
**2**	29^**2**^	7.9	Not detected	30.0	23.4	25.2	15.8	Regional lymph node	Regional lymph node	3	2
**3**	6^**2**^	0.8	Not detected	Not detected	Not detected	13.5	4.1	Distant lymph node	Distant lymph node	3	3
**4**	3^**2**^	2.5	Regional lymph node	Not detected	2.7	9.4	Not detected	Pulmonary nodule	-	1	0
**5**	1^**2**^	7.3	Not detected	21.5	24.3	No metastases	No metastases	-	-	0	0
**6**	2^**2**^	2.7	Not detected	19.0	9.4	No metastases	No metastases	-	-	0	0

*FDG* [^18^F]Fluorodeoxyglucose PET/CT scan; *PSMA* [^18^F]Prostate-specific membrane antigen -1007 PET/CT scan; ^**1**^- FDG was performed before PSMA; ^**2**^ -PSMA was performed before FDG; SUVmax – Maximum Standardized Uptake Value. ^**3**^—Description of the sites detected in each patient:

Patient 1: FDG-PET/CT revealed mesocolic and bilateral external iliac lymph nodes, as well as a lesion in the left pubis, but no uptake in the primary tumor. The mesocolic lymph node showed the highest tracer uptake. PSMA-PET/CT demonstrated mesocolic, external, and internal iliac lymph nodes bilaterally, as well as lesions in the left pubis and ilium, and tracer uptake in the primary tumor. The pubis and left ilium showed the highest tracer uptake

Patient 2: Both FDG-PET/CT and PSMA-PET/CT demonstrated uptake in bilateral external iliac lymph nodes, in addition to the primary bladder tumor

Patient 3: Neither scan showed uptake in the primary tumor. Both tracers demonstrated uptake in superior and inferior paratracheal mediastinal lymph nodes, as well as in right perihilar lymph nodes

Patient 4: FDG-PET/CT revealed a metastatic pulmonary nodule but no uptake in the primary tumor. In contrast, PSMA-PET/CT showed only slight uptake in the primary tumor, with no evidence of metastatic lesions

Patients 5 and 6: Both FDG and PSMA demonstrated uptake in the primary tumors, without evidence of metastatic disease

**Fig. 2:**
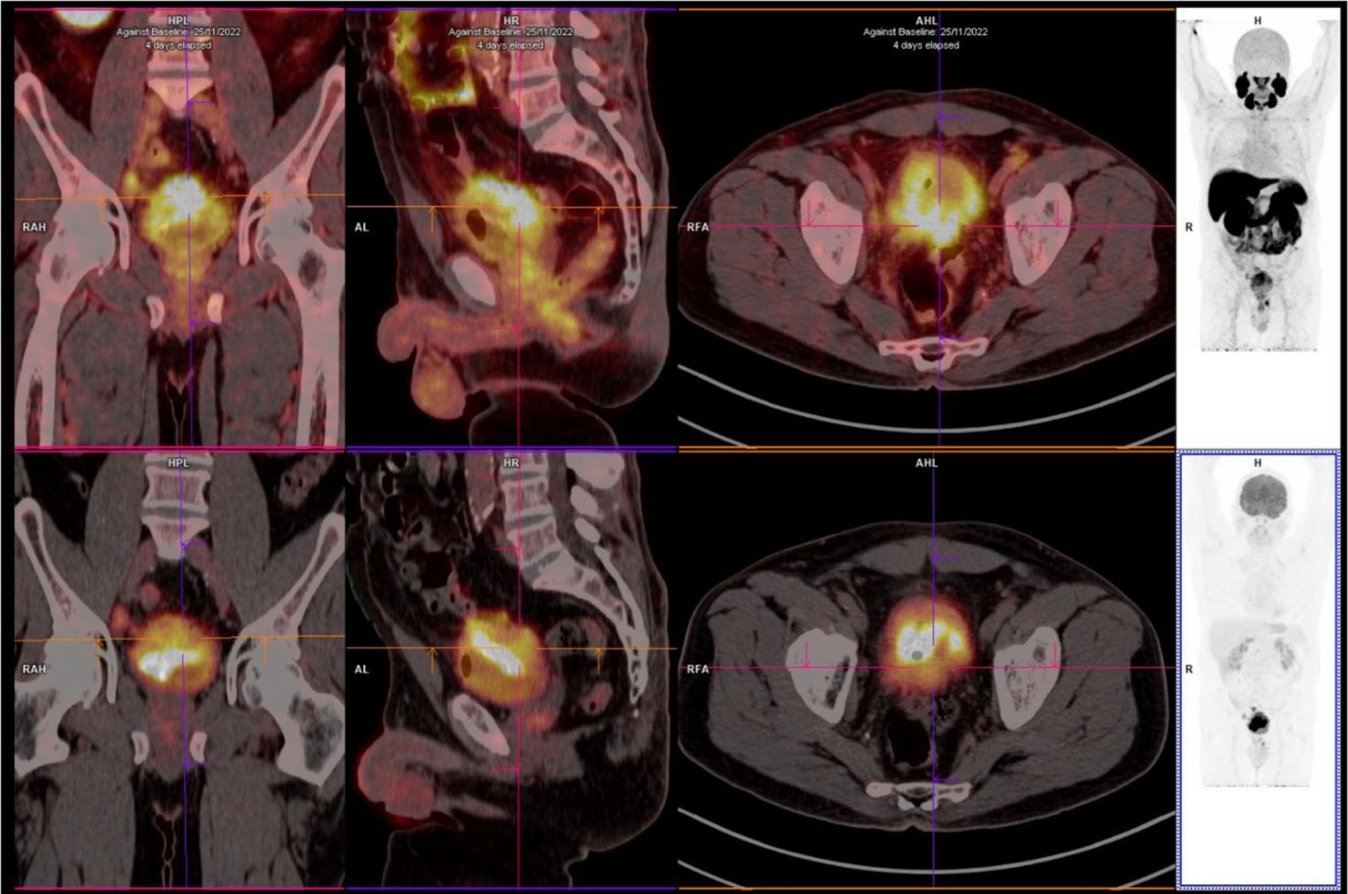
58-year-old, male, T4N1M1, poorly differentiated carcinoma with extensive areas of squamous differentiation. From left to right: coronal, sagittal, axial, and maximum intensity projection [^18^F]PSMA-1007 (top row) and post-diuretic [^18^F]FDG (bottom row) PET/CT images of a patient with muscle-invasive bladder cancer (patient 1, Table 1). Note that an extensive bladder lesion is more clearly identified on [^18^F]PSMA-1007 than on [^18^F]FDG images, in which the lesion cannot be distinguished from urinary excretion of the radiotracer, even after diuretic administration.

[^18^F]PSMA-1007 detected bladder lesions in 5 patients (median SUVmax = 9.8; range: 2.7–24.3) (Fig. 2). [^18^F]FDG detected bladder lesions in only 3 patients (median SUVmax = 21.5; range 19.0–30.0): the bladder lesion was obscured by radioactive urine in one patient, despite diuretic administration, and no [^18^F]FDG activity was identified in a small lesion in another patient. Neither tracer detected active macroscopic lesions in the bladder of one patient.

Both [^18^F]PSMA-1007 and [^18^F]FDG detected regional lymph node metastases in 2 patients (higher SUVmax = 25.2 and 15.8, respectively) and distant lymph node metastases in another patient (higher SUVmax = 4.2 and 13.5, respectively).

Bone metastases were identified in one patient by both [^18^F]PSMA-1007 and [^18^F]FDG (SUVmax = 11.1 and 10.1, respectively), although one additional bone lesion was detected by the former. One patient had an FDG-avid metastasis at the base of the left lung (SUVmax = 9.4), near the spleen, which was not detected by [^18^F]PSMA-1007 due to high physiological uptake in the spleen.

From a clinical perspective, all six patients were diagnosed with MIBC by TUR-BT: five with high-grade urothelial carcinoma and one with poorly differentiated urothelial carcinoma with extensive areas of squamous differentiation. The mean follow-up time was 22.7 months (16–30 months), during which three patients died and three remained alive.

Traditional staging exams (CT/MRI) detected metastases (three lymph nodes and one bone) in four patients. One of these patients presented synchronous involvement of the upper urinary tract, which was not detected by neither [^18^F]FDG nor [^18^F]PSMA-1007 due to physiological uptake by the renal parenchyma and an inflammatory process from a recently performed nephrostomy. These four patients were treated with platinum-based palliative chemotherapy.

Two patients were staged as M0 by conventional staging exams (CT/MRI). One was treated with neoadjuvant chemotherapy followed by radical cystectomy (RC), and the other received trimodal therapy (TUR-BT plus chemo-radiation) [7].

The only patient who underwent RC was the one who had no active lesions detected in the bladder by either tracer. However, histopathological analysis of the surgical specimen showed high-grade invasive urothelial carcinoma classified as pT2a, and pelvic lymphadenectomy was negative for the 16 lymph nodes evaluated, in agreement with PET images. This patient presented positive mediastinal lymph nodes on both the [^18^F]FDG and [^18^F]PSMA-1007 PET/CT images, whose uptake was reduced after neoadjuvant chemotherapy. The patient remained in remission during the 25 months of follow-up.

## Discussion

In MIBC, clinical to pathologic stage discrepancy is relatively common. Postoperative pathologic upstaging occurs in 20–25% of cases staged by conventional imaging (CT/MRI) [8, 9]. On the other hand, the use of [^18^F]FDG PET/CT (adopted by current guidelines [10, 11]) to assess lymph node status upstages patients in about 20% of cases [11] in the locoregional context. Therefore, a more accurate staging examination for this pathology is still required.

### Current Staging Exams

CT remains widely available and is routinely used for initial whole-body staging of bladder cancer, predominantly for nodal and distant metastasis assessment. For local T-staging (assessing the depth of wall invasion), CT is limited by poor soft-tissue contrast and cannot reliably distinguish between lamina propria invasion and muscularis propria invasion in many cases. Therefore, CT is unreliable in differentiating between stages Ta to T3a tumors, although it is useful for detecting invasion into the perivesical fat (T3b) and adjacent organs. For nodal staging, CT relies on size and morphology criteria. Reported accuracy for the detection of locally advanced tumors or nodal metastasis is variable, with studies showing moderate accuracy (for example, sensitivity/accuracy for nodal disease often < 70%, with substantial false negatives for normal-sized metastatic nodes). Thus, CT provides reasonable specificity for bulky nodal disease but limited sensitivity for micro metastatic or small metastatic nodes [12–14].

Magnetic resonance imaging has superior soft tissue contrast resolution compared with computed tomography and can evaluate post-biopsy reactions, as enhancement of the tumor occurs earlier than that of the normal bladder wall due to neovascularization. Multiparametric imaging standardized by the VI-RADS scoring system has substantially improved noninvasive local (T) staging of bladder cancer. Multiple meta-analyses and prospective studies have demonstrated that mpMRI with VI-RADS exhibits high performance in predicting muscle invasion, with pooled sensitivities and specificities often reported in the range of 80–90% (depending on the VI-RADS threshold used and reader experience). VI-RADS also improves inter-reader agreement relative to non-standardized MRI readings and is increasingly recommended for preoperative assessment and for response assessment after neoadjuvant therapy. Both CT and MRI may be used for assessment of local invasion by T3b disease, or higher, but they are unable to accurately diagnose microscopic invasion of perivesical fat (T2 vs. T3a) [15–17].

[^18^F]FDG PET/CT has an established role in whole-body staging because of its ability to detect distant metastatic disease; however, its role in locoregional staging requires careful consideration. Systematic reviews and meta-analyses looking at [^18^F]FDG PET/CT for pelvic lymph-node staging in bladder cancer report moderate diagnostic accuracy with relatively low pooled sensitivity (50% in some pooled analyses) but high specificity (> 90%), meaning that [^18^F]FDG PET/CT is reasonably specific for positive nodal disease but misses a substantial fraction of nodal metastases, particularly small or micrometastatic nodes. [^18^F]FDG PET/MRI hybrids have shown promising results for primary tumor detection and improved soft-tissue delineation in small series and may enhance diagnostic performance for local tumor assessment compared with PET/CT in some analyses. However, urinary excretion of [^18^F]FDG and post-TUR-BT inflammation are practical limitations that can obscure or mimic disease within the bladder and adjacent tissues. Overall, [^18^F]FDG PET/CT is more useful for detecting occult distant metastases and for post-TUR-BT restaging [18, 19].

The strengths and weaknesses of each current bladder cancer staging method are summarized in Table 3 [12–20].

**Table 3:** Strengths and weaknesses of current bladder cancer staging exams [12–20]

Modality	Primary staging objective	Diagnostic performance	Strengths	Key limitations
Multiparametric MRI [15–17]	Differentiate NMIBC (T1) from MIBC (T2)	Pooled sensitivity 83% and specificity 90% for prediction of muscle invasion	Superior soft-tissue contrast; standardized acquisition and reporting (VI-RADS); independent predictor of muscle invasiveness	Best cut-off thresholds still under investigation; affected by post-TUR-BT inflammation; not yet standard of care in all settings
Contrast-enhanced CT [12–14]	Assess locally advanced disease (≥ T3b) and adjacent organ invasion	Unreliable for differentiating Ta–T3a stages; accuracy improves in advanced extravesical disease	Wide availability; rapid acquisition; whole-body coverage	Cannot detect microscopic perivesical fat invasion (T2 vs T3a); limited soft-tissue contrast
CT / MRI for lymph nodes [12–17]	Evaluate pelvic and abdominal lymph node involvement	Sensitivity low (48–87%); specificity also limited due to benign nodal enlargement	Standard cross-sectional assessment; size-based criteria	Unable to identify metastases in normal-sized or minimally enlarged nodes
18F-FDG PET/CT [18–20]	Detection of nodal and distant metastases; treatment planning impact	Low sensitivity and high specificity for nodal metastases; up to 20% of micrometastases missed	May upstage ~ 20% of patients and alter treatment decisions	Urinary FDG excretion; limited sensitivity for microscopic nodal disease; not justified for routine use in cN0 MIBC

*CT* computed tomography; *FDG *fluorodeoxyglucose; *MRI *magnetic resonance imaging; *NMIBC *non-muscle-invasive bladder cancer; *MIBC *muscle-invasive bladder cancer; *TUR-BT *transurethral resection of bladder tumor; *VI-RADS *vesical imaging-reporting and data system

### Current Evidence of [^18^F]PSMA-1007 PET/CT

Recent histological studies have shown that PSMA is expressed in the vasculature of bladder cancer [21–23]. Li et al. evaluated PSMA expression in tissue from TUR-BT or RC of patients with MIBC and identified a correlation between the degree of PSMA expression and other clinicopathological characteristics, such as metastasis, clinical stage, invasive stage, and Ki-67 index [24]. Schreiber et al. also evaluated immunohistochemical PSMA-staining in 89 urothelial cell carcinoma samples and identified a correlation with disease progression, but not with recurrence [25]. In this way, PSMA emerges as a potential predictive or prognostic biomarker in bladder cancer, and cases that capture it more intensely may have characteristics that have not yet been identified, making them potential candidates for theranostic treatment.

Other studies were performed comparing PSMA and FDG PET/CT. Campbell et al. reported a prospective series of three patients and concluded that [^18^F]PSMA PET/CT provided adequate uptake for the detection of primary and metastatic lesions of urothelial carcinoma (UC), although FDG PET/CT had greater ligand uptake across lesions [26]. Lin et al. also compared PSMA and FDG PET/CT, with FDG PET/CT being more effective in detecting lymph node lesions [27]. On the other hand, in a retrospective analysis, Kudachi et al. reported moderate to high-grade 68 Ga-PSMA PET/CT expression when used for the surveillance of histologically proven UC, identifying multiple soft tissue, nodal, and skeletal metastases more effectively than FDG PET/CT [28]. Importantly, none of these studies used [^18^F]PSMA-1007, which improves the evaluation of the urinary tract due to the radiotracer’s predominant biliary excretion.

To the best of our knowledge, our study is the first to provide a comparative analysis of [^18^F]FDG and [^18^F]PSMA-1007 PET/CT in muscle-invasive bladder cancer (MIBC), highlighting the advantages and limitations of each radiotracer in detecting primary tumors, regional lymph node involvement, and distant metastases. The findings suggest that while both tracers demonstrate similar sensitivity for metastatic lesions, [^18^F]PSMA-1007 offers distinct advantages in evaluating primary bladder tumors due to its lower urinary excretion.

One of the biggest challenges of [^18^F]FDG PET/CT for MIBC imaging is the intense urinary elimination of [^18^F]FDG, which can obscure bladder lesions and limit accurate tumor delineation. Despite diuretic administration and delayed imaging, this issue persisted in two patients, leading to under-detection of bladder tumors with [^18^F]FDG. In contrast, [^18^F]PSMA-1007 shows predominant hepatobiliary clearance [29], demonstrating superior lesion visualization within the bladder, identifying lesions in five patients compared to only three with [^18^F]FDG. These findings reinforce the potential PSMA-targeted imaging and theranostic roles in MIBC, especially for primary tumor assessment.

Both radiotracers demonstrated comparable efficacy in detecting regional lymph node metastases, with three patients showing FDG-avid and PSMA-avid nodal involvement. The uptake values (SUVmax) were variable but overlapping between the two tracers, suggesting that both can be used effectively for nodal staging. However, one patient presented an FDG-avid pulmonary metastasis that was not detected by [^18^F]PSMA-1007. This probably occurred because of the proximity between the lesion and the spleen, an organ with high physiological [^18^F]PSMA-1007 uptake. In fact, other organs present limited PET/CT lesion detectability because of physiological tracer uptake, such as the liver for [^18^F]PSMA-1007 and the brain for [^18^F]FDG [30].

A key finding of our study is the substantial uptake of [^18^F]PSMA-1007 in certain MIBC lesions, suggesting potential theranostic implications. PSMA-targeted radionuclide therapy has been increasingly used in prostatic malignancies, and there are ongoing studies in multiple non-prostatic neoplasms, including patients with bladder cancer. The selectivity of PSMA-based ligands for tumor neo-vasculature suggests their potential as valid targets for vascular agents in cancer treatment [31]. Our results indicate that selected MIBC patients with high PSMA expression might benefit from such approaches. However, further investigations, including histopathological correlation and larger patient cohorts, are necessary to validate this hypothesis.

Despite the promising findings, our study has many limitations. The sample size was relatively small. Biopsies of all affected sites would have been very informative, but they were not possible for ethical reasons. The only patient who underwent RC presented with positive histopathological results in the bladder (classified as pT2a), indicating a false-negative result for both [^18^F]FDG and [^18^F]PSMA-1007. In contrast, all resected lymph nodes in this same patient were negative, in agreement with both PET exams. Most patients did not have an indication for radical cystectomy; therefore, confirmation of the metastatic nature of the labeled lesions was based on the evolution of the lesions in imaging exams performed throughout follow-up. Positive mediastinal lymph nodes, as detected by both [^18^F]PSMA-1007 and [^18^F]FDG, appeared to respond to neoadjuvant chemotherapy; however, false-positive results of both tracers due to inflammatory lymph nodes in resolution cannot be excluded. Future studies should include larger cohorts with histological validation to determine the precise role of [^18^F]PSMA-1007-targeted imaging and therapy in MIBC.

Studies involving [^18^F]FDG PET/CT and [^18^F]PSMA-1007 PET/CT are expensive. Therefore, this pioneering real-world study was conducted with a small cohort, aiming to represent a feasibility study. The analysis of this small cohort is fundamental for the design of larger studies. The proposed strategy has potential theranostic implications for immediate clinical implementation, particularly in translating the context of prostate cancer to bladder staging and treatment.

### Future Perspectives

Larger cohorts with histological validation are needed to determine the precise role of [^18^F]PSMA-1007 imaging and therapy in MIBC. The results of the present study could potentially be enhanced by employing PET/MRI instead of PET/CT. PET/MRI combines the molecular imaging strengths of PET with the superior soft-tissue contrast and multiparametric functional capabilities of MRI, thereby improving the detection and characterization of bladder lesions, particularly in situations where urinary tracer excretion compromises accurate tumor visualization. This modality is especially advantageous for pelvic malignancies, where precise anatomical detail is critical, as highlighted in a recent international consensus guideline [32]. Accordingly, the use of [^18^F]PSMA-1007 with PET/MRI may further improve the diagnostic performance of the tracer and should be explored in future studies to validate and expand upon the findings of the present work.

The significant PSMA uptake observed in certain MIBC lesions in this study suggests promising theranostic applications, expanding the role of PSMA-targeted strategies beyond prostate cancer. In particular, the intense uptake of [^18^F]PSMA-1007 in primary bladder tumors and metastatic sites highlights the potential for using PSMA as a diagnostic and therapeutic target in selected patients with MIBC.

PSMA-targeted radionuclide therapy, such as 177Lu-PSMA, has already demonstrated clinical benefit in prostate cancer and is being explored in other PSMA-expressing malignancies, such as urothelial carcinoma. Because PSMA expression in bladder cancer appears to be linked to tumor neo-vasculature rather than the tumor cells themselves, this may offer a therapeutic avenue with less off-target toxicity.

Our real-world findings support the rationale for further investigation of PSMA-based theranostic approaches in MIBC, particularly for patients demonstrating high tracer uptake. Future studies integrating PSMA PET imaging with radionuclide-targeted therapy may help define a new precision medicine strategy for a subset of bladder cancer patients.

## Conclusion

This study provides the first prospective comparison between [^18^F]FDG and [^18^F]PSMA-1007 PET/CT in patients with muscle-invasive bladder cancer (MIBC), highlighting the roles of these radiotracers in staging and disease characterization. The [^18^F]PSMA-1007 PET/CT provides superior visualization of primary bladder tumors compared to [^18^F]FDG, owing to its minimal urinary excretion.

Both tracers exhibit similar sensitivity for lymph node metastases, while [^18^F]FDG may be more reliable for detecting certain distant lesions. The significant [^18^F]PSMA-1007 uptake in some MIBC lesions supports the evidence that [^18^F]PSMA-1007 expression may serve not only as a diagnostic biomarker but also as a potential therapeutic target in urothelial carcinoma. Overall, [^18^F]PSMA-1007 PET/CT imaging represents a promising tool for more accurate staging and personalized management of MIBC.

In accordance with the CONSORT extension recommendations for pilot and feasibility trials, the present real-world findings inform refinements in future study procedures, logistics, and methodological design, which are essential for minimizing research waste by identifying potential problems early, enabling refined interventions, enhanced study rigor, and more effective clinical practices before launching a full-scale, costly trial.

## Data Availability

The datasets used and/or analyzed during the current study are available from the corresponding author on reasonable request. The original contributions presented in this study are included in the article/supplementary material. Further inquiries can be directed to the corresponding author(s).
